# Empowering local leaders in flood inundation mapping in Bagelen, Purworejo, Central Java

**DOI:** 10.4102/jamba.v14i1.1298

**Published:** 2022-08-31

**Authors:** Santika Purwitaningsih, Junun Sartohadi, Lufti Muta’ali, Apolonia D. S. da Costa

**Affiliations:** 1 Department of Disaster Management, The Graduate School, Universitas Gadjah Mada, Yogyakarta, Indonesia; 2 Department of Soil, Faculty of Agriculture, Universitas Gadjah Mada, Yogyakarta, Indonesia; 3 Department of Development Geography, Faculty of Geography, Universitas Gadjah Mada, Yogyakarta, Indonesia; 4 Centre of Geography Study and Spatial Planning, Faculty of Geography, University of Porto, Porto, Portugal

**Keywords:** disaster management, flood inundation, hazard, participatory mapping, reliability

## Abstract

This aricle discusses the reliability of flood inundation information that is obtained from participatory mapping. The commonly applied method to map flood inundation requires both direct and interpretive measurement data based on remote sensing images. Such assessments have limited availability of data; as a result, participatory mapping has become the solution. A number of studies have conducted participatory mapping to obtain flood hazard information in areas with limited sources of data, however, there has been little discussion about its reliability. This research conducted participatory flood inundation mapping by involving local leaders as respondents. The mental map drawn by the local leaders was digitised to obtain a shapefile format map. The information obtained from the semistructured interview was then included in the geographic information system (GIS) data as attributes. The obtained information was compared with the field data to determine its quality. A literature study was then conducted to discuss how the participatory mapping could support managing a disaster. Information obtained through participatory mapping can be effectively applied to disaster management because of its precise location information, lower cost and less time-consuming nature. The reliability of the information has weak accuracy of quantitative data; however, it has advantages in terms of qualitative data, especially in the detailed descriptions of flood information. In the future, participatory mapping should rely on integrating the perspectives of cross-disciplinary researchers, a comprehensive study of multidisciplinary knowledge and level of understanding of the stakeholders.

## Introduction

Information on flood inundation that is important in disaster management is flood extent, flood depth, flood duration and time of occurrence, especially if it occurs in the coastal alluvial plain area. Some flood information for disaster management could be derived from modelling techniques (Thapa et al. 2020:1–12; Tu et al. 2020:1–13; Van Den Bout & Jetten 2020:1–9); however, this method requires a series of data that is measured for a long period (Ahmadalipour & Moradkhani 2019:1–13; Fernandez et al. 2016:265–280; Shrestha et al. 2020:1–17). Not all watersheds in Indonesia have accurate measurement data to be able to conduct flood hydrology models (Fulazzaky 2014:2000–2020); furthermore, flood duration data could not be derived from modelling processes (Singh et al. 2020:1035–1046; Unduche et al. 2018:1133–1149).

The participatory approach is mostly used to collect flood hazard information such as real-time measurement data of flood inundation events and supporting data required for quantitative hazard assessments in areas with limited data (Bizimana & Schilling 2010:99–126). Participatory mapping is an approach to obtain information based on local knowledge by involving local communities in its implementation (Obermeyer 1998:65–66). This approach can involve the community directly (Kienberger 2014:269–275; Singh 2014:161–173; Sudaryatno, Awanda & Pratiwi 2017:1–8) and use the community leaders as the people’s representation (Chingombe et al. 2015:1029–1040). Participatory mapping is mostly performed using traditional methods such as a printed map (Kienberger 2014:269–275; Singh 2014:161–173; Sudaryatno et al. 2017:1–8) and modern information system technology (Koski et al. 2021:1347–1365; Pocewicz et al. 2012:39–53).

## Research area and methods

### Research area

**FIGURE 1:**
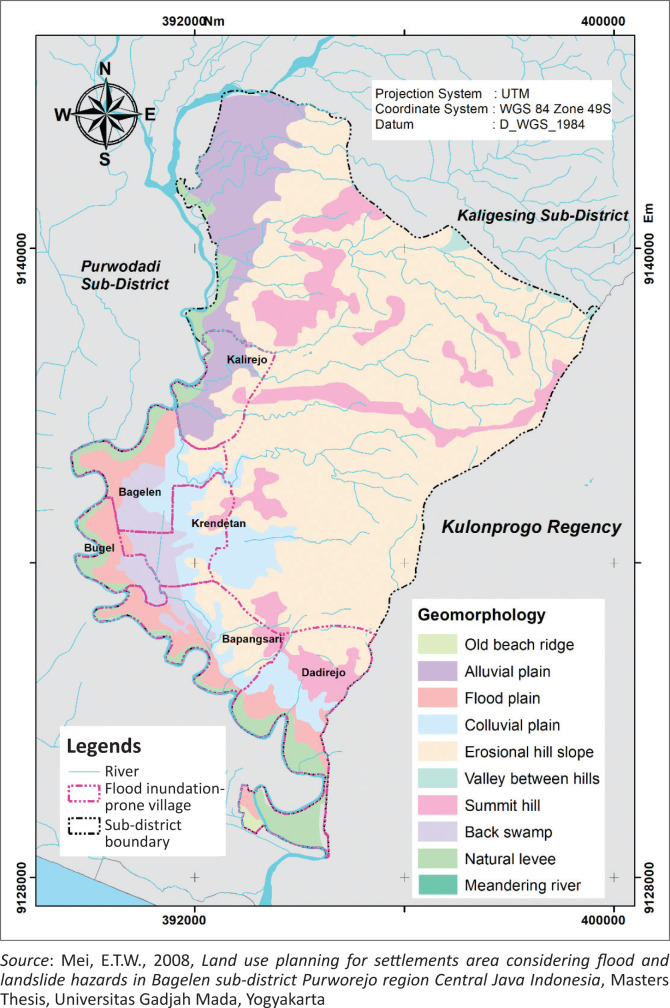
Geomorphology and flood inundation-prone villages of the Bagelen subdistrict (Mei 2008).

The research was conducted in the Bagelen subdistrict of Purworejo Regency, Central Java, Indonesia. The Bagelen subdistrict has a humid tropical climate with an average monthly rainfall of 376.6 mm per month (Purworejo Regency Statistical Center Agency 2019). The Bagelen subdistrict has reliefs of hills and plains, including fluvial, denudational and fluviomarine landforms (Mei, Sudibyakto & Kingma 2009:71–91) (Figure 1). The research area consists of six flood inundation-prone villages: Dadirejo, Bapangsari, Bugel, Krendetan, Bagelen and Kalirejo (Figure 1).

The land use of the flood inundation-prone villages of the Bagelen subdistrict are dominated by crop fields and settlements (43.96% and 38.99%, respectively). Forty-four per cent of the total population in the Bagelen subdistrict are in this flood inundation-prone area; therefore, the people in the area are used to flooding. Several flood mitigations measures have also been conducted by the local community, such as raising the foundations of houses, elevating livestock vehicles and increasing the number of floors of the houses, as well as adding an attic for shelter and emergency storage when a flood inundation occurs. The existing structural flood mitigation measures include drainage system networks, flood walls and gabions.

### Methods

The participatory mapping method and semistructured interview was conducted in March 2020 to collect flood inundation hazard information in the Bagelen subdistrict. During the rainy season, from October 2020 until April 2021, there was no new information related to flood inundation in the study area.

In simple terms, participatory mapping is defined as an approach to obtain information based on local knowledge by involving the local community in its process (Obermeyer 1998:65–66). In a more advanced definition, participatory mapping is considered a method that can change the traditional mapping paradigm from one that seems ‘top down’ to a new mapping paradigm that is able to accommodate everyone’s interests (Goodchild 2007:8–10). In recent years, most participatory mapping activities have been classified into six categories based on their purposes, such as disseminating information to outsiders, recording and storing local knowledge, planning land use and managing resources, promoting changes, increasing community capacity and resolving local conflicts (Corbett 2009). The participatory mapping carried out in this study emphasised the purpose of recording and storing local knowledge.

There are some data required to provide an overview of the problems and conditions associated with the research location before conducting participatory mapping (Bizimana & Schilling 2010; Kienberger 2014:269–275; Sy et al. 2016), such as:

1. Aerial photographs of villages in the Bagelen subdistrict obtained from Google Satellite with a scale of 1:5000.

2. A contour map with 1 m intervals obtained from the processing result of a digital elevation model (DEM) using advanced land-observing satellite (ALOS) phased array type L-band synthetic aperture radar (PALSAR) in 2011 with a resolution of 12.5 m × 12.5 m.

### The selection of the respondents

The respondents were selected through a purposive sampling technique performed prior to the participatory mapping process. Indigenous people with deep knowledge of the area became our criteria in selecting respondents. The respondents involved in this study were local leaders from village areas where flood inundation regularly occurs. The local leader (head of the subvillage) was chosen to be the respondent with the assumption that they are highly knowledgeable in the area. According to Purworejo Regency Regional Regulation Number 7 of 2008, the head of the subvillage has a working period until they are 60 years old.

**FIGURE 2:**
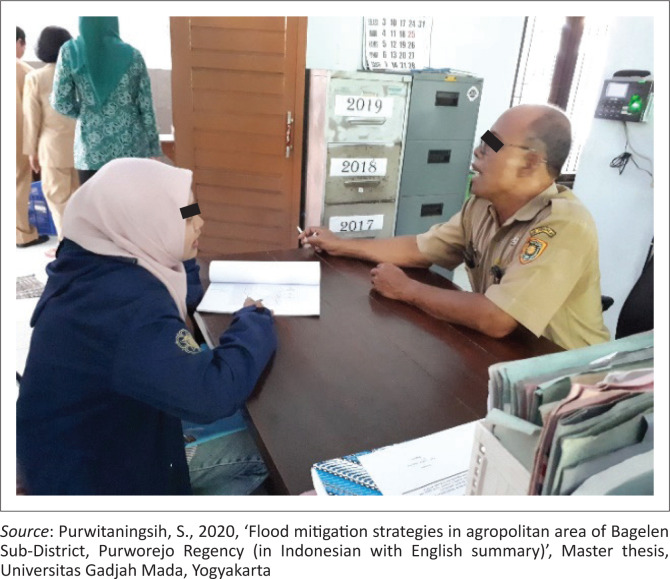
Confirmation with the head of planning, evaluation and general affairs of the Bagelen subdistrict regarding flood-prone villages.

The selection process of the respondents was started by collecting information about the flood inundation-prone villages. Information on the flood inundation-prone villages in the Bagelen subdistrict was collected through confirmation of a macroscaled map of the areas by the head of planning, evaluation and general affairs of Bagelen subdistrict (Figure 2).

Based on the confirmation from the head of planning, evaluation and general affairs of the Bagelen subdistrict, there were six villages prone to flood inundation. Twenty-two out of the 29 subvillages in flood inundation-prone villages of Bagelen subdistrict experienced flood inundation every year; therefore, the heads of the 22 subvillages become our respondents.

### Data collecting process through participatory mapping

**FIGURE 3:**
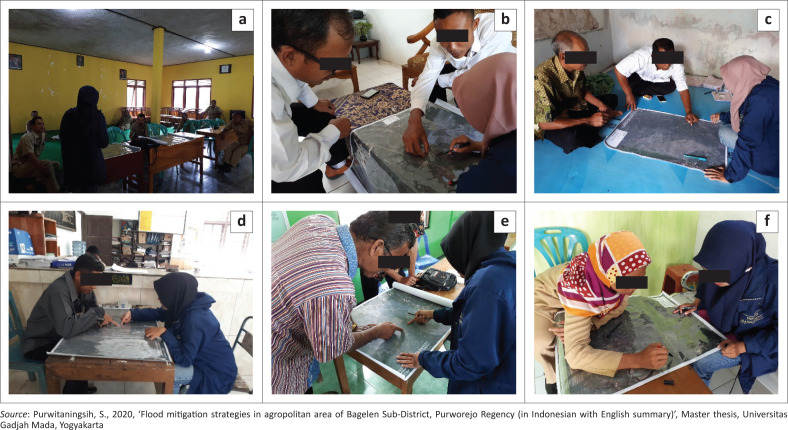
Participatory geographic information system in (a) Dadirejo, (b) Bapangsari, (c) Bugel, (d) Krendetan, (e) Bagelen and (f) Kalirejo villages.

The base map for the participatory mapping process was produced by overlaying aerial photographs of the villages prone to flood inundation with a scale of 1:5000 and the contour map derived from a DEM. The aerial photographs were interpreted to obtain information on elements at risk of flooding. The information on elements at risk of flooding was not only utilised to collect information on flooding areas but also to gather evidence of flooding (by flood marks imprinted on a resident’s house wall, for example). The local respondents then made a map on the base map (containing information on contours and elements at risk) of the flood inundation hazards in their working area (Figure 3). A paper-based participatory mapping was chosen in order to acquire more intensive participation from the respondents (Aditya 2010:119–147).

The maps drawn by the local leaders from their experience were then digitised into a shapefile. Information about the characteristics of the flood inundation was used as attribute data on the map created by the local leaders.

**FIGURE 4:**
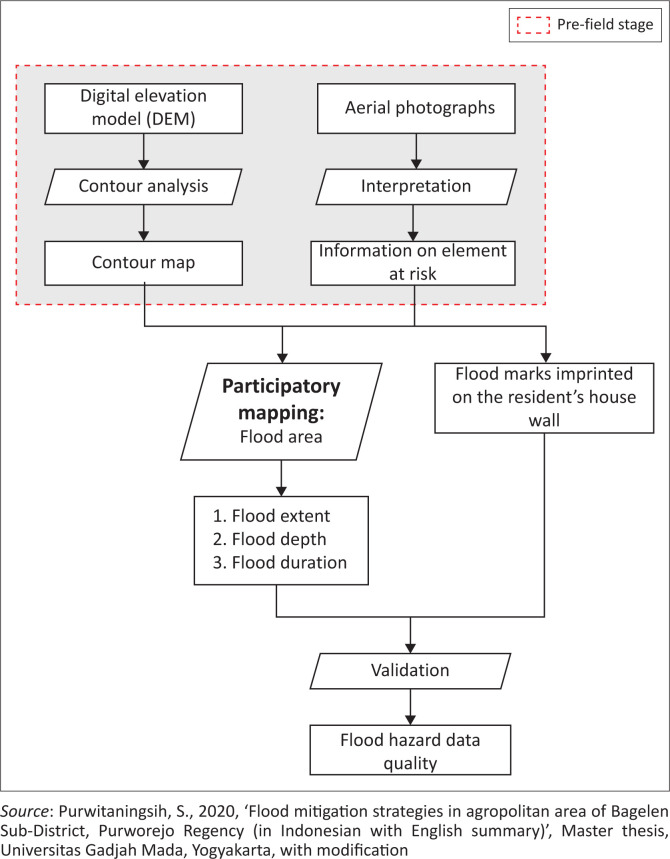
Flowchart of flood inundation participatory mapping.

The flood inundation map was generated based on the worst flood inundation event that occurred in the study area with the assumption that future floods will occur in a similar location place. Semistructured interview techniques were also conducted to obtain qualitative data on the flood inundation characteristics. The flow of the research method used is shown in Figure 4.

### Reliability assessment

To determine the reliability of the participatory mapping data obtained, a comparative and descriptive qualitative analysis was performed to determine the quality of the data. The quality of the participatory mapping-derived data consists of positional (geographic or geometric), attribute, conceptual and logical accuracy and precision variables (Devillers & Jeansoulin 2006:31–42; Shekhar & Xiong 2007). In this article we will only assess positional (geographic or geometric) accuracy and attribute accuracy.

The positional accuracy was determined by studying the literature on the geometric accuracy of the base maps used in the study. The attribute accuracy assessment was conducted by comparing the data obtained from respondents with the data collected in the field. The attribute accuracy assessment was carried out only on the flood depth parameter because the accuracy and precision of the flood extent parameter can be obtained through the assessment of geometric accuracy and precision, whilst the attribute accuracy assessment on the flood duration attribute was not conducted because there was no real-time flood duration data available as the control data.

Flood depth information was collected through flood marks that are still imprinted on local residents’ houses. Thus, the sample used is a resident’s house that still has signs of flood inundation in 2019. From these criteria, 47 samples were obtained to assess the accuracy of the flood depth data. After the quality of the data generated from participatory mapping was ascertained, the reliability of the data on disaster management based on the existing literature was discussed.

### Ethical considerations

The study was conducted with the approval from the Dean of Faculty of Agriculture, Universitas Gadjah Mada (reference number: UN1/PSASDL/TU/LL/2022). This study has followed the provided guidelines for study procedures.

## Results

### Respondents

The respondents in this study were the heads of flood inundation-prone subvillages of the Bagelen subdistrict (22 respondents, Table 1), the heads of flood prone villages (six respondents) and an official from the government of the Bagelen subdistrict (one respondent), chosen through a purposive sampling technique. All the local leaders involved in this study were younger than 60.

**TABLE 1:** List of subvillages affected by flood inundation.

Number	Village	Subvillage
1	Dadirejo	Karangjambu, Kuwojo, Jurangkah and Karangnongko
2	Bapangsari	Joho, Sangkalan, Bapangan, Srapah, Pucungan, Bojong and Sudimoro
3	Bugel	Sembir and Bugel
4	Krendetan	Semawung
5	Bagelen	Kalidiren, Segeluh, Kalibelung, Bedug, Gatep and Bagelen
6	Kalirejo	Keposong and Kahuripan

*Source*: Purwitaningsih, S., 2020, ‘Flood mitigation strategies in agropolitan area of Bagelen Sub-District, Purworejo Regency (in Indonesian with English summary)’, Master thesis, Universitas Gadjah Mada, Yogyakarta

### Flood inundation in the Bagelen subdistrict

Flood inundations occur every year in the Bagelen subdistrict during the peak of the rainy season. The largest flood inundation event ever in the Bagelen subdistrict was used as a reference in collecting flood inundation information and mapping the flood inundation hazards, with the assumption that future flood inundation events will occupy the areas hit by this event. Based on semistructured interviews conducted with the local leaders, the largest flood inundation events in the Bagelen subdistrict occurred in 2019 (Dadirejo, Bapangsari, Bugel, Krendetan, Bagelen village) and 2016 (Kalirejo village). The flood inundation in 2019 was ascertained to be larger than the flood inundation in 1965 and similar to the one in 2004. Information regarding the year of the largest flood inundation event is in accordance with the statement of the head of Bapangsari village that the 2019 flood inundation was the largest event after the flood inundations in 1965 and 2004:

‘[*R*]elated to floods, this area is sure to experience flooding every year, ma’am. But last year’s flood was the most intense. Since my childhood till now, the largest floods were the 1965, 2004 floods and last year’s floods, ma’am.’ (Local leader, Male, Bapangsari Village)

Information about the largest flood inundation events in 2019 and 2016 was also confirmed by the statement of the head of Bagelen village that in the 2019 and 2016 flood inundations, the high flood level caused water to enter the building:

‘[*T*]he largest flood that happened here was last year’s flood, ma’am. The water gets into the office here. Usually, the water just reaches the yard.’ (Local leader, Male, Bapangsari Village)

The shapefile of the largest flood inundation distribution that occurred in the Bagelen subdistrict was obtained by digitising the map made by the local leaders. The area affected by the largest flood inundation in the study area reached 856.63 ha or 44.83% of the area of the flood inundation-prone villages (Table 2).

**TABLE 2:** The subvillage area affected by the flood inundation.

No.	Village	Area (ha)	Area affected by flood inundation (ha)	Percentage (%)
1	Dadirejo	539.55	109.81	20.35
2	Bapangsari	368.21	192.64	52.32
3	Bugel	151.20	151.20	100.00
4	Krendetan	345.82	237.61	68.71
5	Bagelen	253.02	96.84	38.27
6	Kalirejo	252.84	68.53	27.10
Total		1910.64	856.63	44.83

*Source*: Purwitaningsih, S., 2020, ‘Flood mitigation strategies in agropolitan area of Bagelen Sub-District, Purworejo Regency (in Indonesian with English summary)’, Master thesis, Universitas Gadjah Mada, Yogyakarta

### Flood inundation hazard characteristics

It is important to know the characteristics of the flood hazard to select future mitigation measures. Information on the characteristics of the flood inundation hazards in the Bagelen subdistrict was obtained through participatory mapping completed with semistructured interviews.

The flood that occurs every year including the largest flood in 2016 and 2019 were an overflow of the Bogowonto River and its tributaries, which are upstream of the Bagelen subdistrict. A flash flood has never occurred in the Bagelen subdistrict. This information was obtained from the statement of the head of subdivision of planning, evaluation and general affairs of Bagelen subdistrict:

‘[*H*]ere, the floods are usually due to Bogowonto, ma’am. It is also caused by the trapped rainwater that cannot reach into the channel. There has never been a flash flood here.’ (Government official, Male, Bagelen subdistrict)

**FIGURE 5:**
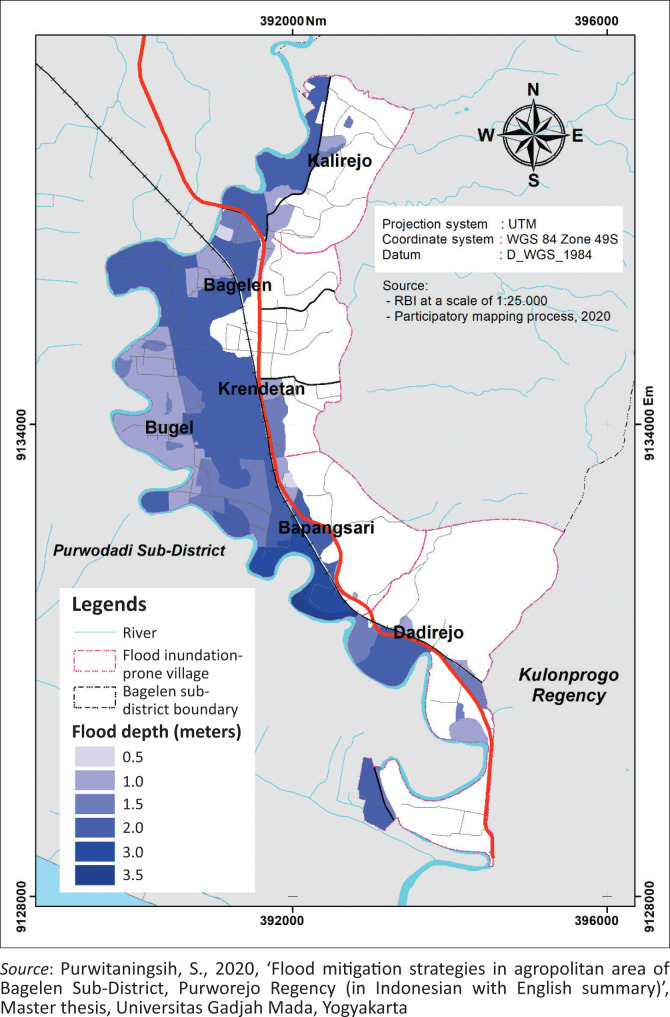
Flood depth map.

The flood depth information was collected through a map drawn by local leaders of the Bagelen subdistrict. The map was also accompanied by qualitative data obtained from the interview. Most of the respondents mentioned the flood depth using their own measurements, such as ankle-depth, knee-depth, waist-depth, windowsill-depth, to the roof of the house and even more. However, we then tried to ask more about their perception of the flood depth using metres. Based on the perceptions of the local leaders, the depth of the largest flood inundation that occurred in the Bagelen subdistrict varied from less than 0.5 m to more than 3.5 m. This flood depth range was found throughout the area of the Bagelen subdistrict. However, the deepest flood depth was found in the Bapangsari village (more than 3.5 m). The local leaders said that the depth of the flood inundation reached the roof of the house. In Dadirejo village, the southernmost village of the Bagelen subdistrict experienced a depth of approximately 0.5 m – 2 m (the flood depth varied from knee-depth to the upper wall of the house). Bugel village experienced a flood depth of approximately 1 m – 1.5 m (the flood inundation varied from waist-depth to windowsill-depth). The Krendetan and Kalirejo villages experienced a depth of approximately of 0.5 m – 2 m (the flood depth varied from ankle-depth inside the house until it reached the roof of the house). In Bagelen Village, the flood depth was approximately 0.5 m – 2 m (varied from knee-depth to the upper wall of the house). The flood depth map of Bagelen subdistrict is exhibited in Figure 5.

The largest flood inundations in the Bagelen subdistrict had a duration of less than 24 h. Kalirejo village, Bagelen village and some parts of Krendetan village experienced flooding for no more than 6 h. This information is in accordance with the statement of the leaders of Kahuripan and Kalibelung subvillage:

‘[*T*]he largest flood in 2016 was about half a day, ma’am; then it was receded.’ (Local leader, Female, Kahuripan subvillage)

‘[*T*]he last flood didn’t take long time, ma’am; it took about 3 or 4 h and then continued to recede.’ (Local leader, Male, Kalibelung subvillage)

Some parts of Krendetan village specifically located near Bugel village experienced flood inundation for up to 12 h:

‘[*T*]he flood around the paddy fields area was longer; it was almost all day, ma’am.’ (Local leader, Male, Semawung subvillage)

Most of Bapangsari village experienced flood inundation for up to 24 h. The western part of Bapangsari village experienced flood inundation for 24 h, whilst the eastern part experienced flood inundation with shorter duration. This is in accordance with the information provided by the head of Bapangan subvillage. The subvillage itself is located in the western part of Bapangsari village:

‘[*I*]n this area, the floods took longer, ma’am. In the morning, around half past 8, the water started to enter the village; then it subsided only the next morning. It was about 23 h or more.’ (Local leader, Male, Bapangan subvillage)

‘[*A*]round 7 pm, the water started to rise; then around 7 am, it receded, ma’am.’ (Local leader, Male, Bapangan subvillage)

**FIGURE 6:**
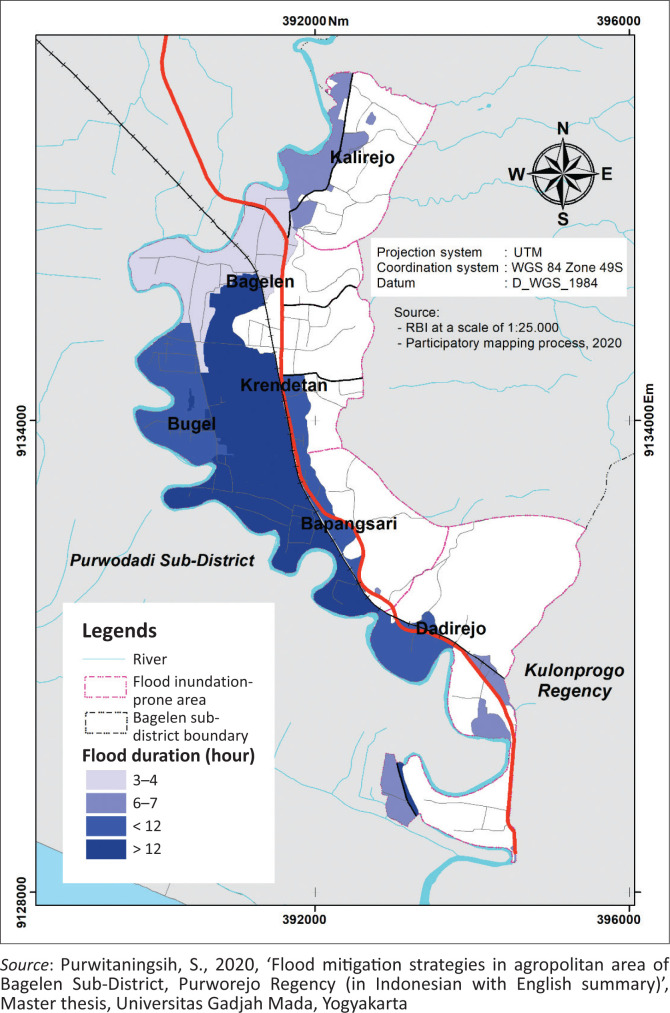
Flood duration map.

Information about the flood duration is presented in spatial form in Figure 6.

## Discussions

### The quality of data obtained through participatory mapping

Data quality could be defined as the degree of how well data can serve its specific purpose. High-quality data can represent reality accurately. Data quality has many dimensions, such as accuracy, accessibility, completeness, consistency, integrity, validity and currency (Batini & Scannapieca 2006). In this research, the determination of data quality was based on its accuracy and accessibility. Accuracy shows the degree of the information depicted on a map matched with the information in the real world (Shekhar & Xiong 2007). In the geographical and geospatial domain, the accuracy of a map consists of positional accuracy and attribute or thematic accuracy. Positional accuracy indicates the accuracy of geographical position (horizontal and vertical accuracy), and attribute or thematic accuracy presents the value accuracy of the properties (Batini & Scannapieca 2006).

It is important to compare the data collected through participatory mapping with the data directly collected from the study area to know its accuracy. However, from three parameters (flood extent, flood depth and flood duration), there will only be one hazard parameter to be compared with the data from the field study, the flood depth.

The validation of the flood extent parameter was not conducted because of the high accuracy of the instrument utilised in the participatory mapping process and the high data confidence by the respondents involved. The base map for participatory mapping was created from aerial photographs and a contour map of the Bagelen subdistrict. The aerial photographs were from Google Satellite, which has geometric accuracy that meets the accuracy standard of a Class 3 base map at 1: 5000 scale (Akbar 2018). The contour map is the result of contour analysis from DEM ALOS PALSAR with a pixel size of 12.5 m × 12.5 m and vertical accuracy of 0.8 m (Julzarika & Dewi 2018:11–24). Both the aerial photographs and contour map used for the base map have high geometry accuracy.

Furthermore, the respondents involved in this research were local leaders from 22 subvillages that are prone to flooding. The local leaders are native persons of the subvillage and based on the local regulation (Regulation of the Government of Purworejo Regency No. 7/2008), the local leaders are supposed to lead the subvillages (which have small areas) until they are 60 years old. Therefore, they have lived in the area for a long time and have rich knowledge of their area, so the level of detail of their description of the location and other flood hazard information is very high (up to the name of the person whose house was affected by the flood and the part of the house that was flooded), representing high data confidence. The exception is also in accordance with the statement of Servigne, Lesage and Libourel (2006:179–185) regarding validation of data through validation of geometric accuracy, where the geometric accuracy could become the validation measure of information accuracy if it is related to a location as an attribute. At this point, flood extent is information related to location. Validation of the flood duration cannot be conducted because of the unavailability of recorded data when the largest flood occurred.

Forty-seven samples were collected during the field study in October 2020 to compare the flood depth information detected in the field with that provided by the local leaders (Table 3). The flood depth samples were collected from the flood marks that were observed in residents’ houses. The field study to compare the flood depth with two different sources was constrained by the condition of residents’ houses that had been repainted, so it was difficult to find traces of flood inundation in some residents’ houses. In addition, the data collection of flood depth in nonresidential areas could not be conducted because of the unavailability of signs of flood marks. The selection of flood marks indicated that the largest flood was obtained from the information of the house owner.

**TABLE 3:** Compatibility assessment of the information obtained from the respondents and the field data.

No.	Code of polygon	Maximum flood depth based on the perceptions of local leaders (m)	Flood depth based on observation (m)	Compatibility
1	KJRT2	1.0–2.0	0.15; 1.78; 1.95	Matched
2	SGRT1	1.5–2.0	1.14; 1.36	Matched
3	BGLRT2	1.5–2.0	2.28	Not matched
4	BDRT1	1.5–2.0	2.00	Matched
5	PSRT1	< 1.0	No data	No data
6	KRRT3	1.0–1.5	0.50; 0.76	Matched
7	KRRT5	1.0–2.0	No data	No data
8	BJRT1	1.0–3.0	1.46	Matched
9	BGRT3	< 1.0	1.43; 1.23; 1.23	Not matched
10	SRRT1	1.5–2.0	1.22	Matched
11	BGRT1	1.0–1.5	No data	No data
12	KHRT3	< 1.0	0.55	Matched
13	KHRT5	1.0–1.5	0.60	Matched
14	PSRT4	1.0–2.0	0.47	Matched
15	PSRT3	0.5	No data	No data
16	BGRT2	1.0–1.5	1.14; 0.28	Matched
17	SBRT2	1.0–1.5	No data	No data
18	SBRT1	1.0–1.5	No data	No data
19	GTRT1	< 1.0	1.67	Not matched
20	KHRT4	< 2.0	0.50; 0.50	Matched
21	PSRT6	1.0–1.5	0.50; 0.55	Matched
22	PSRT2	< 1.0	0.70	Matched
23	KRRT4	1.0–1.5	No data	No data
24	KRRT2	1.0–1.5	No data	No data
25	KRRT1	0.5–1.0	0.43	Matched
26	JHRT1	1.0–2.0	1.86	Matched
27	JHRT2	0.5–1.0	1.30	Not matched
28	JHRT3	1.0–1.5	No data	No data
29	SKRT1	1.0–1.5	No data	No data
30	BPRT2	2.0–3.0	No data	No data
31	BPRT3	2.0	1.60; 1.68	Matched
32	BPRT1	1.5	No data	No data
33	SKRT4	1.5–2.0	1.50	Matched
34	SKRT2	1.0–1.2	No data	No data
35	SKRT3	1.5–2.0	1.88	Matched
36	JHRT4	1.0–1.5	No data	No data
37	BPRT4	2.0–3.0	1.46	Matched
38	SDRT2	0.5–1.0	No data	No data
39	SDRT1	1.5–2.0	No data	No data
40	PCRT1	< 2.0	3.39; 1.25	Not matched
41	BGLRT1	< 2.0	No data	No data
42	KBRT1	< 1.0	0.71	Matched
43	KDRT1	< 2.0	1.11	Matched
44	BDRT2	< 0.5	No data	No data
45	BDRT3	1.0–1.5	No data	No data
46	BDRT4	1.0–1.5	No data	No data
47	BDRT5	< 0.5	No data	No data
48	SRRT3	0.2–0.5	1.15	Not matched
49	SRRT2	0.4–1.0	No data	No data
50	SBRT3	< 1.0	No data	No data
51	BJRT2	2.0–3.5	No data	No data
52	SRRT4	0.5–0.75	0.70	Matched
53	SRRT5	1.0–2.0	No data	No data
54	KJRT3	1.0	1.05	Matched
55	SDRT3	< 1.0	No data	No data
56	JRRT3	0.5–1.0	1.13	Not matched
57	JRRT2	1.0–1.2	1.50	Not matched
58	KJRT1	1.0–1.5	1.11; 1.24	Matched
59	KWRT2	1.0–1.2	1.02	Matched
60	KNRT4	< 2.0	No data	No data
61	KNRT2	0.5–1.0	No data	No data
62	KWRT1	< 1.0	No data	No data
63	KNRT3	1.5	0.28; 1.09	Matched
64	BGRT4	1.5–2.0	No data	No data

The flood depth data collected through the participatory mapping does not have high accuracy (Table 3) because of bias in converting the flood depth perceptions of respondents into quantitative data. Most of the respondents mentioned the flood depth in their measurements, such as ankle-depth, knee-depth, waist-depth, windowsill-depth or to the roof of the house. The perception of each respondent about the exact size of the measures is different, which causes many differences in the quantitative measurements of flood depth between the information from the respondents and the data obtained in the field. This finding is the same as that of Musungu (2015), who has mentioned this regarding the results from participatory mapping, which are mostly in the form of public perceptions with various measurements that are more qualitative than quantitative data. However, qualitative data obtained from participatory mapping has high accuracy because the respondents are people who have lived in the area for a long time, so they have rich knowledge of the conditions of the area.

The hazard map generated from participatory mapping has high geometric accuracy and precision and high attribute precision regarding more qualitative data. However, because the respondents were mostly given information based on their perceptions and their measurements, there would be many biases that occur during the conversion of their measurements into standard measurement. As a result, the participatory mapping does not have high accuracy, specifically regarding quantitative data.

### Participatory mapping in disaster management

The purpose of disaster management is to protect the community from the threat of disasters and ensure the implementation of a planned, integrated, coordinated and comprehensive disaster management. Based on Republic of Indonesia Law No. 24 of 2007, disaster management has the principles of being fast and precise, high-priority, coordinated and integrated, efficient and effective. Thus, the data that are obtained quickly and accurately can play an important role in preventing the occurrence of higher numbers of victims and damages because of disasters. In this context, the data obtained through the participatory mapping process has several advantages over data obtained through other processes.

Flood inundation hazard mapping through participatory mapping has a better level of effectiveness and efficiency when compared with other methods or approaches. The effectiveness and efficiency of a mapping method can be determined by measuring the time and cost involved in its process. The less time and cost required, the more effective and efficient it is. The time and costs required to carry out participatory mapping are relatively small compared with mapping using other approaches that are usually carried out over a large area and require a lot of data and high expertise in its process (Calianno, Ruin & Gourley 2013:1–13; Choi, Kang & Kim 2021:1–10; Spitalar et al. 2014:863–870). The time required for participatory mapping consists of time for preparing a base map and time for conducting participatory mapping of respondents. The time needed to collect data as a base map is relatively short because it only involves collecting secondary data. The amount of time required to conduct a participatory mapping depends on the number of respondents involved. Respondents involved in the study were selected using the purposive sampling technique, so there were not many respondents involved, considering the small area of the research. The small number of respondents means that the flood inundation hazard mapping through participatory mapping did not take much time. Flood inundation hazard mapping through participatory mapping also does not require a high level of expertise, so the process is relatively easy and does not take much time. The costs required in the participatory mapping process only include the cost of transportation to the respondent and preparing a base map for the participatory mapping, which include the cost of printing base maps and purchasing stationery.

Mapping with a participatory mapping approach uses local people’s knowledge as a source of information, so it is suitable for small areas. Mapping in the small area usually has a large scale, which means that it also has detailed information (Butler et al. 1987). Large-scale data have advantages when applied in disaster management, which aims to reduce casualties as much as possible to the individual unit. Moreover, as it directly involves the community or community representatives who have the potential to be exposed to disasters as the source of information, a map derived from participatory mapping has better accuracy for vulnerability assessment in disaster management (Liu et al. 2018:1–23).

The map generated from participatory mapping has high geometric accuracy and precision. It can be seen from the base map utilised and the respondents involved in creating the map. Maps that have high geometric accuracy and precision have insignificant differences in the location from the actual conditions (Shekhar & Xiong 2007). A hazard map that has high geometric accuracy and precision means that the hazard distribution in the location depicted on the map is indeed likely to occur in the actual position. When linked with disaster management, hazard maps with high geometric accuracy and precision will be very beneficial in determining areas prone to disasters, so a great number of victims and losses because of disaster can be reduced.

Apart from its advantages, the hazard maps generated from the participatory mapping do not have high attribute accuracy, specifically regarding the magnitude of the hazard. Furthermore, in case of flood hazards, participatory-based maps have no information about water velocity. Thus, when implemented in disaster management activities, they cannot be used as reference data in terms of quantitative assessments, so they must be supplemented by maps generated through other, more quantitative approaches.

### Local leaders’ empowerment for flood disaster

Strengthening, protective and preventive measures are the most important goals for disaster risk issues that require integrated and participatory management. Anticipating the risk of social vulnerability, local leaders become actors or sources of valid information for capacity building through practical participatory actions, that is, disaster mapping. Indonesia has a comprehensive disaster management regulation, but unfortunately there is no preview of future solutions to the turmoil of deadly natural disasters that frequently happen at almost all rural and urban regions and islands in Indonesia. To deal with this nonprovidable regulation, the European Union (EU) has just recently established the Recovery and Resilience Facility, which involved putting into effect a systematic and integrated 36 articles of the 8 chapters of regulation on 12 February 2021, where the recovery and resilience scoreboard in Article 30 put forward its long-term anticipation, impact and management and community involvement and participation for the disaster (The European Parliament and the Council of the EU 2021). In the context of the long-term sustainability of community-centred disaster management, the role of participatory mapping cannot be separated from the guidance of practitioners and scientists of physical geography and social geography to carry out joint and continuous assessments. This study involves several experts (authors themselves) in the study of the application of participatory practical methods for impact analysis and also sociogeographic management in nontechnical or substantive qualitative engagements, which are relatively simple but included the trajectory of participatory social understanding. The trajectory of social understanding in geography itself refers to the sociogeographic understanding that preventive measures are carried out through the provision of accurate information from trusted sources, namely local leaders. This understanding and their all roles refer to the context of social geography, which is approachable and relevant to its implementation of risk communication between local leaders, local communities and practitioners and scientists or geographers.

In risk communication, the relationship between people represents a social understanding or theory. Social understanding is a term that draws attention to the embeddedness of intellectuality in social life and contexts that shape our local knowledge, however imperiously global their claims to know, and to the practical consequences of understanding (and indeed being in) the world like this rather than like that (Gregory 1994). Local knowledge values a cohesive and cooperative disaster management team in response to risk quantity and people’s space of living when a disaster occurs, in this case flooding in the research location. Thus, social understanding towards risk communication amongst the local leaders, practitioners and geographers is a continuous series that has a fairly dynamic status quo or varies in respondents’ perceptions of diversity for obtaining a new knowledge of disaster risk management. Geography brings together people, places and communication to solve risk or vulnerability to disaster through a variety of practical approaches, that is, participatory mapping. This was confirmed by previous authors who proposed a new method for defining community that views geography on a continuum and suggest that membership within a community is moderated by place (Carbone & McMillin 2018:121–133). This kind of disaster management is the anticipation or preparedness and earlier thematic planning of flooding and solutions for the long run in the future. It is closely related to the daily context of local people’s lives, actions that are implemented in adapting and inputs inspired during the disaster adaptation process. Furthermore, it also refers to research on communities and their resilience or adaptive capacities, processes or empowerments in dealing with floods, a contextual example of a problem in Timor Island, Indonesia (Da Costa 2018). Past flood events both locally and globally have meaningful insight into the experience of the people for future adjustment. It is commonly referred as adaptive capacities - their ability to learn from past experiences and adjust themselves to future challenges in their everyday lives (Keck & Saldaporak 2013:5–19). Previous studies have also suggested that the adjustment process in human geography perspective is taken by:

[*T*]he role of human mobility for participatory informants that research works on human mobility for geographical studies and urban planning whose goal is to find people’s important places of movement and, specifically, to analyse their mobility between home and work with the purpose of location tracking. (Ebrahimpour et al. 2020:1–33)

We believe that the global goal for the future management of disaster movement is a sense of initiating some future norms of adaptive management.

The study of disaster management from traditional to modern methods and vice versa is not merely a discipline but an expanded discipline along with the systematic resilience of the community, as perceived implicitly by a Mexican social scientist (Solis-Gadea 2006:113–122).

## Conclusion

The information on flood inundation obtained from participatory mapping is available on a large scale because it is obtained from a small area. The detailed information from large-scale maps is very relevant to applications in disaster management, which has the principle of reducing casualties to the individual units. Detailed information about flood inundations obtained through participatory mapping does not take much time and cost, so it is very suitable to be applied in disaster management, which generally requires accurate data in a short time. Although the information on flood inundation obtained through participatory mapping has high geometric accuracy, it does not have high accuracy regarding the magnitude of the hazard in a quantitative way, including flood velocity, so supplementary data would be needed to be applied in further assessment for disaster management. In the future, it is recommended that the participatory mapping process should focus on integrative analysis that relies on qualitative data rather than quantitative data of the element at risk. Elements of disaster risk need to be reviewed from a cross-disciplinary researcher’s point of view with a mixed method of qualitative and quantitative and a comprehensive study of social geography, especially the application of mitigation, understanding or education related to disasters and disaster resilience at local, regional and international scales, from and for public (social) safety, by involving stakeholders. This is expected to be a coherent and useful review or input for the progress of the processes of implementing the planning and sustainability of disaster management in Indonesia in the future and future global adjustment norms for disaster.

Importantly, it would be more suitable as an integrative assessment and a malleable approach towards combined participatory approach and community participation in disaster management analyses if a specific physical geography analysis on the element of risk and vulnerability assessment is applied for future studies. This is due to the fact that the respondent involved has the potential to be directly exposed to disasters. Research in the future should be expanded periodically to prevent obsolescence of the meaning and application of the GIS technology and supporting statistics method used.
